# Public policy on breastfeeding among working mothers in Taiwan: comparison between two national surveys

**DOI:** 10.1186/s12884-023-06069-4

**Published:** 2023-11-03

**Authors:** Pei-Jung Yu, Wan-Ru Wu, Chieh-Yu Liu

**Affiliations:** 1 Mackay Medical College, New Taipei City, Taiwan, R.O.C.; 2 Tzu Chi University, Hualien City, Taiwan R.O.C.; 3 National Taipei University of Nursing and Health Science, Taipei City, Taiwan R.O.C.

**Keywords:** Breastfeeding, Lactation room, Working mothers, Returning to work

## Abstract

**Background:**

Creating a supportive breastfeeding environment after childbirth and enabling women to work with reassurance are essential in maternal care services. The study aimed to explore the effectiveness of the utilization rate of public and workplace lactation rooms in relation to the breastfeeding rate among postpartum women returning to work in Taiwan.

**Methods:**

The study involved a secondary data analysis on 6,992 and 7,350 postpartum women surveyed in 2011 and 2016, respectively. Interviews were conducted with women six months postpartum. Logistic regression analysis was employed to calculate the odds ratio and investigate the differences in the utilization rates of public lactation rooms (PLR) and workplace lactation rooms (WLR) among working mothers over a five-year period, to confirm the effectiveness of public strategies.

**Results:**

Comparing the years 2011 and 2016, significant differences in the rates of exclusive breastfeeding (EBF) and any breastfeeding (ABF) among working mothers returning to work after an 8-week maternity leave, depending on whether they used PLR or WLR. The rates were higher in 2016 than in 2011. For mothers who used PLR, the breastfeeding rates for EBF at the second, fourth, and sixth months (2011 vs. 2016: 67.6% vs. 81.1%, 75.0% vs. 86.4%, 77.5% vs. 86.2%) and ABF at the second, fourth, and sixth months (2011 vs. 2016: 60.3% vs. 73.9%, 68.8% vs. 81.3%, 73.7% vs. 85.6%). For mothers who used WLR, the breastfeeding rates for EBF at the second, fourth, and sixth months (2011 vs. 2016: 51.3% vs. 58.7%, 54.7% vs. 61.4%, 57.5% vs. 59.3%) and ABF at the second, fourth, and sixth months (2011 vs. 2016: 48.4% vs. 57.0%, 52.3% vs. 60.5%, 54.1% vs. 62.4%). When comparing 2011 to 2016 from the second to the sixth month postpartum, adjusted odds ratios for EBF (PLR: 4.17-5.23 vs. 4.06-6.22, WLR: 1.71-1.83 vs. 1.30-1.61) and ABF (PLR: 6.44-7.02 vs. 9.27-9.90, WLR: 1.91-1.98 vs. 1.97-1.99) showed differences.

**Conclusion:**

Lactation support rooms play a vital role in motivating working mothers to sustain breastfeeding upon their return to work. Incentivizing businesses to build additional lactation rooms and offering breastfeeding resources is essential in striving to enhance the global breastfeeding rate.

## Background

Breastfeeding support is an important public health responsibility. In recent years, breastfeeding-friendly workplace environmental policies for working mothers have become key health issues [1]. The World Breastfeeding Week (WBW) emphasizes that breastfeeding support is a primary priority. Improving breastfeeding policies should include lactation rooms and other infrastructures in the workplace. There are three Sustainable Development Goals (SGDs) related to breastfeeding support that are endorsed by the World Health Assembly (WHA). These goals include employers supporting and meeting the needs of breastfeeding women in the workplace (SDG 8), reducing poverty and disadvantaged groups’ job inequality (SDG 10), and making mothers in big cities feel safe and welcome to breastfeed in public places (SDG 11) [2]. Therefore, positive operational improvement or enhancement of policies supporting breastfeeding is important to urgently increase friendly breastfeeding environments.

Empowering working mothers positively impacts child and maternal nutrition and health [3]. Mothers returning to work after maternity leave require adequate breastfeeding breaks, lactation support facilities, and leadership strategies [4]. A systematic review showed that breastfeeding at work is not only possible but also more likely when employers provide the support women need [5]. Insufficient lactation rooms and places to store breast milk result in limited lactation capacity, which makes mothers feel guilty and affects their mental health [6, 7]. Several studies have confirmed that women have acknowledged the importance of environmental resources in supporting lactation. The difficulty in setting up nursing rooms is influenced by financial, cultural, and political factors. It also requires the government and employers to work together [8]. The nutritional, physical, and mental health of postpartum women and their babies depend on systematic administration policies for breastfeeding support, especially for postpartum working mothers. Meeting all prerequisites for promoting breastfeeding among working mothers will enable adequate well-being for both mothers and infants. It can also create sustainable health promotion for WBW- SDG activities. The sufficient lactation rooms and complete breastfeeding facilities in the workplace can enable postpartum women to accomplish their natural breastfeeding tasks.

Global gender equality can be achieved through women’s employment. Research on breastfeeding working mothers is abundant, but there is an urgent need to explore employers’ experiences and perspectives to improve the effectiveness of breastfeeding support [9]. Australia used citizen science to engage the public in monitoring workplace breastfeeding support. They pointed out that, while workplace support varied widely, women often reported having to use poorly equipped and/or unhygienic communal spaces to breastfeed or express milk at work. These findings raise important questions about the interpretation and implementation of current legislation to support working women [10]. Employers are willing to pay attention to the importance of breastfeeding for working mothers, and providing a high-quality breastfeeding environment should be able to create a win-win situation. This enables the company to improve business operations, and female employees and their families to obtain a better state.

Over the past 20 years, there have been improvements in women’s education levels and an increase in employment opportunities in the service industry in Taiwan. From 2003 to 2012, the average percentage of postpartum working mothers breastfeeding during their 56-day maternity leave rose from 66.9–87.6%. The average percentage of women who continued to breastfeed after returning to work also increased from 10.6–24.1% [11]. The employment growth rate of married women was significantly higher than that of all working women at 39.69% [12]. Taiwan is continually advancing in promoting breastfeeding support strategies due to health professionals’ emphasis on the importance of breastfeeding for the health of both the mother and the baby. In 2014, the Department of Labor Standards and Equal Employment under the Ministry of Labor created breastfeeding policies under the Gender Work Equality Act. Employers must provide eight weeks of maternity leave for childbirth. If an employee needs to breastfeed or collect milk for a child under the age of two, the employer should add 60 minutes daily, in addition to regular breaks. If work is extended by over an hour, 30 minutes of breastfeeding time is required, and breastfeeding time counts as working hours. Companies with over 100 employees must provide a breastfeeding room, which can be subsidized by authorities [13].

The author underscores the importance of promoting a conducive breastfeeding environment, which is being actively done in many countries. The World Health Organization (WHO) recommends exclusive breastfeeding for the first 6 months for infants. Nonetheless, many regions face difficulties in attaining these recommended breastfeeding rates, particularly concerning women reintegrating into the workforce after childbirth. Therefore, we advocate for an analysis of the utilization rates of public and workplace lactation rooms among women returning to work more than 6 months after giving birth, with a particular focus on sustaining breastfeeding. Despite the establishment of breastfeeding policies in Taiwan, including the 2014 Gender Equality in Work Act, which aimed to benefit working mothers, challenges persist. Given that Taiwan's standard maternity leave is 8 weeks, we explored the related factors and effectiveness of breastfeeding support for working women after they return to work at the 2nd, 4th, and 6th month postpartum. The findings of this research are anticipated to provide invaluable insights to working women and employers alike, shedding light on the significance of sustaining breastfeeding and the subsequent adoption of comprehensive breastfeeding support services.

## Methods

### Reserch design

This study employed a cross-sectional survey research method using secondary data analysis from two national surveys of postpartum women commissioned by the Taiwan Health Promotion Administration in 2011 and 2016. The purpose of this study was to use a survey data of utilization rates of public and workplace lactation rooms among women returning to work at the second, fourth, and sixth months postpartum to compare the effects of lactation support facilities on breastfeeding. Subsequently, this research aims to effectively address the challenges and disparities in breastfeeding support for working mothers, with the overarching goal of enhancing maternal and child health outcomes.

### Participant

The main purpose of this study was to estimate national postpartum women’s use of breastmilk-friendly environmental resources, such as the breastfeeding rate and whether they used lactation rooms in public places or workplaces. A systematic sampling method was used to randomly select participants from 22 Taiwanese cities. Participants in the telephone interview had to be > 20 years old and > 6 months postpartum. In surveys of 12455 and 12536 women in 2011 and 2016, working mothers accounted for 56.4% (*N* = 6992) and 58.8% (*N* = 7350), respectively. The cohort of working mothers analyzed in this study were mothers who were more than 6 months postpartum, and they were full-time workers at the time of the interview. While the specific return date to their workplace was not queried, it was verified that each of them had availed themselves of an 8-week maternity leave. The main reasons for not completing the questionnaire were unanswered calls, participants not being at home, and refusal to participate. We compared the sample with national statistics on women who gave birth in the indicated years and found no significant differences in regional distribution.

### Instrumets

A structured questionnaire was conducted via telephone interviews with postpartum women. The questionnaire was tested using an expert validity method. Experts in the field of breastfeeding and representatives from the National Health Service were invited to participate. The content validity meeting was chaired by the administrator and held at the Health Promotion Administration of the government. Finally, a questionnaire was developed by researchers and experts.

### Data collection procedure

First, we applied for the release of Institutional Review Board (IRB) and birth notification data. Trained investigators used the Computer-Assisted Telephone Interview System to conduct the interviews. Second, interviewer and supervisor training were conducted, and the interviewees were notified of the scheduled time for the telephone interviews. After receiving a phone call and obtaining informed consent, the data were collected using a structured questionnaire.

Women recalled their breastfeeding history from their hospital stay to six months postpartum. Enquiry about each item in the breastfeeding support environment was done by asking questions. Background characteristics included sociodemographic factors, such as maternal age, maternal education level, occupation, and parity.

### Statistical analysis

Descriptive results were reported as frequencies and percentages for categorical variables. The relationship between working mothers and housewives was assessed using a logistic regression model. Cross tables, chi-square, and binary logistic regression were used to yield crude and adjusted odds ratios (ORs) using the public lactation room (PLR) and workplace lactation room (WLR) of working mothers. Missing variables were excluded from the data analysis because they accounted for < 2% of the total. All statistical analyses were performed using IBM SPSS Statistics 24.0.

## Results

Postpartum working mothers in 2016, compared with those in 2011, tended to be older, more educated, and earn more income. In 2016, the rate of rooming-in increased (3.5% vs. 19.3%) and early skin-to-skin contact decreased (56.3% vs. 37.3%). Postpartum women had more contact with peer supporters (3.5% vs. 55.5%), breastfeeding buddies (16.9% vs. 52.7%), and lactation consultants (14.2% vs. 54.5%) and had a higher use of public (49.0% vs. 64.1%) and workplace lactation rooms (43.7% vs. 53.2%) than in 2011 in Table 1.

**Table 1:** Characteristics of working mothers of this study

	2011 (*N* = 6992)		2016(*N* = 7350)	
	working mothers	%	working mothers	%
Maternal age				
20–24	268	3.8	363	4.9
25–29	1580	22.6	1512	20.6
30–34	3368	48.2	3363	45.8
> 35	1776	25.4	2112	28.7
Education level				
lower than secondary	1541	22.0	1320	18.0
secondary education	1596	22.8	879	12.0
higher than secondary	3853	55.1	5140	69.9
Parity				
1	3822	54.7	4037	54.9
2	2654	38.0	2747	37.4
3	456	6.5	505	6.9
≥ 4	57	0.8	60	0.8
Income				
low income	257	3.7	179	2.4
lower-middle income	1129	16.1	1213	16.5
upper-middle income	3618	51.7	3759	51.1
high income	1290	18.5	1554	21.1
Rooming in				
yes	248	3.5	1418	19.3
no	6947	96.5	5392	80.7
Early skin to skin				
yes	3935	56.3	2740	37.3
no	2968	42.4	4481	61.0
Peer supporters				
yes	248	3.5	4080	55.5
no	6745	96.5	3270	44.5
Breastfeeding volunteers				
yes	1180	16.9	3870	52.7
no	5813	83.1	3479	47.3
Lactation consultant				
yes	994	14.2	4005	54.5
no	5998	85.8	3344	45.5
Using PLR				
yes	3425	49.0	4709	64.1
no	3567	51.0	2641	35.9
Using WLR				
yes	3050	43.7	3913	53.2
no	3931	53.6	3400	46.3

*PLR* Public lactation room, *WLR* Workplace lactation room

Table 2 presents the results of EBF comparison between 2011 and 2016, except for the 6th month (23.5% vs. 13.3%), all other time points are higher in 2016 than in 2011. The total breastfeeding (ABF) rates in 2016 were all higher than that for in 2011 during the hospital stay (90.3% vs. 87.5%), the first month postpartum (92.2% vs. 90.0%), the second month postpartum (81.5% vs. 75.2%), the fourth month postpartum (66.9% vs. 58.9%), and the sixth month postpartum (55.8% vs. 48.7%). In 2011 and 2016, the EBF remained at 50% in the 2nd month after delivery and gradually declined from the 4th to the 6th month. However, in 2016, the ABF rate remained above 55.8% (55.8%-90.3%) until the sixth month after delivery.

**Table 2:** Exclusive and any breastfeeding rate among working mothers

	2011(*N* = 6992)		2016(*N* = 7350)	
	working mothers		working mothers	
**Exclusive breastfeeding**				
EBF in hospital	3220	46.1	2257	30.7
EBF at 1 month	4240	62.1	4245	58.1
EBF at 2 months	3435	50.3	3691	50.5
EBF at 4 months	2588	37.9	2939	40.2
EBF at 6 months	1607	23.5	969	13.3
**Any breastfeeding**				
ABF in hospital	6120	87.5	6638	90.3
ABF at 1 month	6089	90.0	6757	92.2
ABF at 2 months	5089	75.2	5974	81.5
ABF at 4 months	3987	58.9	7899	66.9
ABF at 6 months	3293	48.7	4088	55.8

In 2011 and 2016, the rates of EBF and ABF in working mothers who used public and workplace lactation rooms were significantly higher than that for in working mothers who did not (*p* < 0.001) in Table 3. The 2011 postpartum EBF and ABF rates of women who used vs. did not use PLR in the second, fourth, and sixth months can be found in Table 4 under the adjusted OR. Table 4 shows that the adjusted ORs of EBF and ABF of working mothers who used PLR and WLR were all higher than those of working mothers who did not use PLR and WLR in 2011 and 2016. After the adjusted odds ratio, the use of lactation rooms in 2016 was higher than that in 2011. For used PLR mothers, rates were as follows (2011 vs. 2016): EBF at the second month (67.6% vs. 81.1%), fourth (75.0% vs. 86.4%), and month (77.5% vs. 86.2%); ABF at the second (60.3% vs. 73.9%), fourth (68.8% vs. 81.3%), and sixth month (73.7% vs. 85.6%). For used WLR mothers, rates were: EBF at the second (51.3% vs. 58.7%), fourth (54.7% vs. 61.4%), and sixth month (57.5% vs. 59.3%); ABF at the second (48.4% vs. 57.0%), fourth (52.3% vs. 60.5%), and sixth month (54.1% vs. 62.4%). When comparing 2011 to 2016 from the second to the sixth month postpartum, working mothers who used PRL with those who did not, the adjusted odds ratios of EBF (4.17-5.23 vs. 4.06-6.22); the adjusted odds ratios of ABF (6.44-7.02 vs. 9.27-9.90); working mothers who used WRL with those who did not, adjusted odds ratios for EBF (1.71-1.83 vs. 1.30-1.61); adjusted odds ratios for ABF (1.91-1.98 vs. 1.97- 1.99)

**Table 3:** Rate of using PLR and WLR after returning to work of working mothers after postpartum 2^nd^, 3^rd^, and 6^th^

2011 (*N* = 6992)									2016 (*N* = 7350)							
PLR					WLR				PLR				WLR			
	*N*	Yes(%)	No(%)	*p*	*N*	Yes(%)	No(%)	*p*	*N*	Yes(%)	No(%)	*p*	*N*	Yes(%)	No(%)	*p*
**Exclusive breastfeeding**																
EBF at 2 months	3435	67.6	34.2	0.000	3247	51.3	48.7	0.000	691	81.1	18.9	0.000	3674	58.7	41.3	0.000
EBF at 4 months	2598	75.0	25.0	0.000	2584	54.7	45.3	0.000	2939	86.4	13.6	0.000	2924	61.4	38.6	0.000
EBF at 6 months	1607	77.5	22.5	0.000	1604	57.5	42.5	0.000	969	86.2	13.8	0.000	961	59.3	40.7	0.000
**Any breastfeeding**																
ABF at 2 months	5090	60.3	39.7	0.000	5080	48.8	51.2	0.000	5974	73.9	26.1	0.000	5944	57.0	43.0	0.000
ABF at 4 months	3987	68.8	31.2	0.000	3980	52.3	47.7	0.000	4900	81.3	18.7	0.000	4873	60.5	39.5	0.000
ABF at 6 months	3293	73.7	26.3	0.000	3287	54.1	45.9	0.000	4088	85.6	14.4	0.000	4066	62.4	37.6	0.000

**Table 4:** Odds ratio of using PLR and WLR after returning to work of working mothers after postpartum 2^nd^, 3^rd^, and 6^th^

2011 (*N* = 6992)					2016 (*N* = 7350)			
Crude OR (95% CI)			Adjusted OR (95% CI)		Crude OR (95% CI)		Adjusted OR (95% CI)	
	PLR	WLR	PLR	WLR	PLR	WLR	PLR	WLR
**Exclusive breastfeeding**								
EBF at 2 months	4.45 (4.02–4.92)	1.86 (1.68–2.04)	4.17 (3.76–4.63)	1.71 (1.55–1.89)	4.89 (4.40–5.43)	1.52 (1.39–1.67)	4.76 (4.27–5.31)	1.47 (1.34–1.62)
EBF at 4 months	5.72 (5.13–6.38)	2.04 (1.85–2.56)	5.23 (4.68–5.85)	1.81 (1.63–2.00)	6.59 (5.84–7.43)	1.70 (1.55–1.88)	6.22 (5.50–7.04)	1.61 (1.46–1.78)
EBF at 6 months	4.88 (4.29–5.55)	2.06 (1.84–2.31)	4.39 (3.85–5.01)	1.83 (1.63–2.06)	4.03 (3.34–4.87)	1.31 (1.14–1.50)	4.06 (3.34–4.93)	1.30 (1.13–1.50)
**Any breastfeeding**								
ABF at 2 months	7.37 (6.42–8.47)	2.41 (2.14–2.71)	6.44 (5.59–7.43)	1.98 (1.75–2.24)	10.67 (9.25–12.31)	2.15 (1.91–2.43)	9.90 (8.55–11.46)	1.79 (1.58–2.03)
ABF at 4 months	7.74 (6.92–8.66)	2.37 (2.14–2.63)	7.02 (6.25–7.88)	1.97 (1.77–2.19)	10.32 (9.22–11.55)	2.34 (2.12–2.58)	9.56 (8.51–10.73)	1.99 (1.79–2.20)
ABF at 6 months	7.64 (6.85–8.51)	2.89 (2.08–2.53)	6.83 (6.11–7.64)	1.91 (1.72–2.11)	10.06 (8.99–11.20)	2.27 (2.07–2.50)	9.27 (8.27–10.40)	1.97 (1.78–2.17)

Adjusted for maternal age, educational level, and parity. *p* < .001

## Discussion

Our study indicates that working mothers have outnumbered housewives in the past decade. In recent years, working mothers have used public and workplace lactation rooms more frequently, and have had better contact with peer supporters, breastfeeding buddies, and lactation consultants.

The EBF rate in our research shows that postpartum women returning to the workplace after the sixth month decreased; however, in the second month, it was approximately 50%, which is similar to the third-month postpartum rate of 51.1% in Japan [14]. However, one study found that for well-educated, socially advantaged, and highly motivated working mothers, exclusive breastfeeding for up to six months is a challenge due to the difficulty in finding a balance between being a good worker and a good mother. The author believes that it is unrealistic for all women to undergo EBF for six months and suggests that this period should be adjusted from person to person [15]. In another qualitative study, most participants reported that supervisors’ attitudes toward breastfeeding were insufficient to promote EBF. Mothers’ attitudes, workplace, and employment conditions, as well as support received for breastfeeding, were the main determinants of EBF duration [16].

Our ABF rates were also similar to those of 14 studies included in a systematic review and meta-analysis, with an overall average prevalence of breastfeeding after women return to work of 25% and ABF rates ranging from 2–61% [1]. In 2016, our study showed that the ABF rate exceeded 55.8% in all months up to the sixth. During the 5-year period from hospitalization to the second, fourth, and sixth months after delivery, we found that the ABF in 2016 in Taiwan increased by 2.4% − 14.6% compared with that in 2011. Previous research and this study have proposed that working mothers are willing to continue exclusive breastfeeding while working. Although the rate of exclusive breastfeeding among working mothers has declined in recent years, overall breastfeeding rates have increased, indicating that working mothers want to continue breastfeeding. However, many factors that become obstacles hinder the promotion of exclusive breastfeeding for at least six months. Policies and facilities for exclusive breastfeeding for working mothers are worth strengthening to help them provide better nutrition to mothers and babies. Ultimately, employers require support to continue this practice.

If the workplace has a dedicated lactation room and maintains a comfortable and clean environment, mothers continue breastfeeding after returning to work [17]. The WHO’s Western Pacific Office research showed that female staff who work there suggested the establishment of lactation rooms, regular bulletins on breastfeeding support policies, and celebrating World Breastfeeding Week annually, which will protect, promote, and support breastfeeding [18]. The availability of lactation rooms in the workplace is one of the important factors affecting the breastfeeding rate of women returning to work after childbirth [19]. In a cross-sectional study in France, hospital departments had strategies to promote continuous breastfeeding, but only 20.4% of lactation rooms (11/54) were equipped to comply with statutory requirements. Almost half of the respondent departments did not provide lactation rooms. They concluded that measures must be taken to promote breastfeeding using effective equipment to allow postpartum women to work comfortably [20]. De Sousa and da Silva [21] point out that extended re-feeding time can improve physical and mental health, allow women to carry out working activities comfortably, and contribute to a good relationship between female employees and employers while practicing SDG 8. Therefore, lactation rooms in workplaces should be encouraged and facilitated. Many previous studies, as well as our results, have shown that lactation rooms in workplaces play a key role in continued breastfeeding among working mothers. In our study, the use of lactation rooms increased in the second, fourth, and sixth months postpartum in 2016 compared to 2011. The rate of exclusive breastfeeding increased by 3.1% -14.4%, and the overall breastfeeding rate increased by 15.3% -16.8%. After adjusted maternal age, educational level, and parity, the OR = 1.79–1.97 (*p* < .001). The establishment of lactation rooms in the workplace plays an important role in the continuous breastfeeding of working mothers.

Lactation rooms in public places are also necessary for breastfeeding mothers who need to move between two places because of work. A previous study has shown that, considering the benefits of long-term breastfeeding as well as the comfort of breastfeeding women and children, it is necessary to set up dedicated breastfeeding places in public. Their results showed that 10% of the 78% of women who used lactation rooms in public places were criticized, and 8.6% never took their babies out because of a lack of suitable places and equipment, feelings of embarrassment, or sympathy from others [22]. A study, nearly 95% of postpartum women believe that lactation rooms should be in public places. They are strongly suggested that breastfeeding support should include better policies of breastfeeding in public spaces as well as increased construction of public lactation rooms [23]. Another similar research showed that 78% − 81% of breastfeeding mothers have breastfed their babies in restaurants and in shopping malls, but for those who want to breastfeed exclusively, despite laws supporting public breastfeeding, attitudes, and spaces for public breastfeeding need to improve [24]. The embarrassment experienced by mothers while breastfeeding in public is often cited as a key factor in their decision to stop breastfeeding. They believe that a movement is needed to view public breastfeeding as normal and desirable, and increasing public lactation rooms may increase social perceptions of public breastfeeding acceptance [25]. A comprehensive analysis of women’s experiences of breastfeeding in public from 27 publications from 12 countries revealed the core themes of enhancement and challenge, confirming the international difficulties that women experience while breastfeeding. A multilevel approach is needed to address community and social behavior issues, and public breastfeeding experiences and facilities need to be enhanced and improved [26]. In several countries, postpartum women face a lack of space for breastfeeding in public places and inadequate policies to support breastfeeding, which affect breastfeeding outcomes. However, our findings indicate that working mothers who used public lactation rooms 2–6 months ABF after delivery were nine (adjusted OR = 9, *p* < .001) times more likely than women who did not use public lactation rooms in 2016. Compared to 2011, there was an increase of 26% in 2016.

In the International Women’s Day, the World Alliance for Breastfeeding Action calls upon organizations to improve the conditions for working women to breastfeed, such as optimal paid maternity/parental leave and workplace support [27]. In late 2022, the president of the United States signed a two-bill law protecting the rights of pregnant workers and nursing mothers. It covers salaried and hourly employees, and the time spent expressing breast milk [28]. And employers with 50 or more employees must provide private spaces for nursing mothers to express breast milk to their babies under the break time for nursing mothers of the Fair Labor Standards Act in the United States [29]. According to the above emphasis policy changes, our research confirms the importance of public lactation rooms for breastfeeding women and shows that the promotion of government policies has had a positive effect since 2014.

## Conclusions

Maternal and infant nutrition, as well as health and breastfeeding outcomes, were evaluated in alignment with the Sustainable Development Goals (SDGs) set forth by the World Health Assembly (WHA) and the World Breastfeeding Week (WBW) initiative. Numerous countries are actively engaged in the development, implementation, and enhancement of breastfeeding promotion initiatives. Our research findings present robust evidence highlighting the pivotal role of lactation rooms in fostering a conducive environment for working mothers to sustain breastfeeding upon their return to work. Furthermore, these findings underscore the significance of proactive and well-directed national strategies in contributing to the attainment of international breastfeeding goals. Governments and businesses should, therefore, adopt and implement policies that promote breastfeeding-friendly workplaces, thus encouraging women to continue breastfeeding. Our results not only facilitate continued breastfeeding for working mothers, thereby benefiting the health of their infants, but also contribute to the effective functioning of economic development. To effectively pursue the global objective of increasing breastfeeding rates, it becomes imperative to incentivize companies to establish additional lactation rooms and provide accessible breastfeeding resources. Moreover, to ensure the effect of investigative measures, longitudinal measures, including tracking the actual outcomes of exclusive breastfeeding, merit further research.

## Data Availability

The data that support the findings of this study are available from Health Promotion Administration, Ministry of Health and Welfare (MOHW) and National Health Research Institutes (NHRI) but restrictions apply to the availability of these data, which were used under license for the current study, and so are not publicly available. Data are however available from the authors upon reasonable request and with permission of Health Promotion Administration, Ministry of Health and Welfare (MOHW) and National Health Research Institutes (NHRI).

## References

[1] Dutheil F, Méchin G, Vorilhon P, Benson AC, Bottet A, Clinchamps M (2021). Breastfeeding after returning to work: a systematic review and meta-analysis. Int J Environ Res Public Health.

[2] World Alliance for Breastfeeding Action (WABA). Breastfeeding & Sustainable Development: WABA has made links between breastfeeding and each of the Sustainable Development Goals (SDGs). https://waba.org.my/. [12/28/2022]

[3] Santoso MV, Kerr RB, Hoddinott J, Garigipati P, Olmos S, Young SL (2019). Role of women’s empowerment in child nutrition outcomes: a systematic review. Adv Nutr.

[4] Hill EK, Bimbi OM, Crooks N, Brown R, Maeder AB (2023). Uncovering the experience: return to work of nurses after parental leave. J Emerg Nurs.

[5] Dinour LM, Szaro JM (2017). Employer-based programs to support breastfeeding among working mothers: a systematic review. Breastfeed Med.

[6] Peters GW, Kuczmarska-Haas A, Holliday EB, Puckett L (2020). Lactation challenges of resident physicians - results of a national survey. BMC Pregn Childbirth.

[7] Edemba PW, Irimu G, Musoke R (2022). Knowledge attitudes and practice of breastmilk expression and storage among working mothers with infants under six months of age in Kenya. Int Breastfeed J.

[8] Fernandes VMB, Santos EKAD, Erdmann AL, Pires DEP, Zampieri MFM, Gregório VRP. Establishment of lactaton rooms in public and private companies: potentialities and difficulties. Rev Gaucha Enferm. 2017;37(spe): e20160044610.1590/1983-1447.2016.esp.2016-0046. Portuguese, English PMID: 28640333. 10.1590/1983-1447.2016.esp.2016-0046 28640333

[9] Chang YS, Harger L, Beake S, Bick D (2021). Women’s and employers’ experiences and views of combining breastfeeding with a return to paid employment: a systematic review of qualitative studies. J Midwifery Womens Health.

[10] Rowbotham S, Marks L, Tawia S, Woolley E, Rooney J, Kiggins E (2022). Using citizen science to engage the public in monitoring workplace breastfeeding support in Australia. Health Promot J Austr.

[11] Chen YC, Wu YC, Chie WC (2006). Effects of work-related factors on the breastfeeding behavior of working mothers in a Taiwanese semiconductor manufacturer: a Ministry cross-sectional survey. BMC Public Health.

[12] Department of Labor Statistics, Ministry of Labor. The Status of Women’s Labor Participation in my country in Recent Years., 2018. Available from: https://www.mol.gov.tw/1607/2458/2464/2474/. [12/28/2022].

[13] Ministry of Labor. What are the regulations on breastfeeding time in the Gender Equality in Work Act?, 2014. Available from: https://www.mol.gov.tw/1607/28690/2282/2284/2320/7368/post. [12/28/2022].

[14] Nakada K (2021). Effectiveness of a breastfeeding program for mothers returning to work in Japan: a quasi-experimental study. Int Breastfeed J.

[15] Alianmoghaddam N, Phibbs S, Benn C. Reasons for stopping exclusive breastfeeding between three and six months: a qualitative study. J Pediatr Nurs. 2018; 39:37–43. 10.1016/j.pedn.2018.01.007. Epub 2018 Jan 19. PMID: 29525214. 10.1016/j.pedn.2018.01.007 29525214

[16] Gebrekidan K, Hall H, Plummer V, Fooladi E (2021). Exclusive breastfeeding continuation and associated factors among employed women in North Ethiopia: a cross-sectional study. PLoS ONE.

[17] Tsai SY. Impact of a breastfeeding-friendly workplace on an employed mother’s intention to continue breastfeeding after returning to work. Breastfeed Med. 2013;8(2):210–6. 10.1089/bfm.2012.0119. Epub 2013 Feb 7. PMID: 23390987; PMCID: PMC3616406. 10.1089/bfm.2012.0119 PMC3616406 23390987

[18] Iellamo A, Sobel H, Engelhardt K (2015). Working mothers of the World Health Organization Western Pacific offices: lessons and experiences to protect, promote, and support breastfeeding. J Hum Lact.

[19] Litwan K, Tran V, Nyhan K, Pérez-Escamilla R (2021). How do breastfeeding workplace interventions work?: A realist review. Int J Equity Health.

[20] Barasinski C, Stankovic M, Debost-Legrand A, Delabaere A, Vendittelli F, Dutheil F (2022). Workplace lactation support: a cross-sectional study in a university hospital and a perinatal network. Nutrients.

[21] De Souza CB, Venancio SI, da Silva RPGVC. Breastfeeding support rooms and their contribution to sustainable development goals: a qualitative study. Front Public Health. 2021; 9:732061. 10.3389/fpubh.2021.732061. PMID: 35004566; PMCID: PMC8733197. 10.3389/fpubh.2021.732061 PMC8733197 35004566

[22] Grzyb J, Grzyb Ł, Wilińska M. Perception and practice of breastfeeding in public in Poland. J Mother Child. 2022;25(4):277–84. 10.34763/jmotherandchild. 20212504. d-21–00020. PMID: 35675812; PMCID: PMC9444198. 10.34763/jmotherandchild.20212504.d-21-00020 PMC9444198 35675812

[23] Zhao Y, Ouyang YQ, Redding SR (2017). Attitudes of Chinese adults to breastfeeding in public: a web-based survey. Breastfeed Med.

[24] Russell K, Ali A (2017). Public attitudes toward breastfeeding in public places in Ottawa. Canada J Hum Lact.

[25] Morris C, Zaraté de la Fuente GA, Williams CE, Hirst C. UK Views toward breastfeeding in public: an analysis of the public’s response to the Claridge’s incident. J Hum Lact. 2016 Aug;32(3):472–80. 10.1177/0890334416648934. Epub 2016 May 18. PMID: 27193432. 10.1177/0890334416648934 27193432

[26] Hauck YL, Bradfield Z, Kuliukas L (2021). Women’s experiences with breastfeeding in public: An integrative review. Women Birth.

[27] World Alliance for Breastfeeding Action (WABA). Working women have the right to support for breastfeeding! March 8, 2023. Available from: https://waba.org.my/working-women-have-the-right-to-support-for-breastfeeding/

[28] Wage and Hour Division, United States Department of Labor. FLSA protections for employees to pump breast milk at Work. Revised 2023. Available from: https://www.dol.gov/agencies/whd/fact-sheets/73-flsa-break-time-nursing-mothers. [5/8/2023].

[29] Congress.gov. Providing urgent maternal protections for Nursing Mothers Act or the PUMP for Nursing Mothers Act, H.R.3110 - PUMP for Nursing Mothers Act, 117th Congress (2021–2022), 2021. Available from: https://www.congress.gov/bill/117th-congress/house-bill/3110. [5/8/2023]

